# Osteoporosis Not Millet-disease

**Published:** 1896-05

**Authors:** T. D. Hinebauch

**Affiliations:** Fargo, N. D.


### CORRESPONDENCE.

### OSTEOPOROSIS NOT MILLET-DISEASE.

Editors of Journal of Comparative Medicine, etc.

After the publication of the article I read at the first meeting in America of the International Veterinary Congress, several criticisms appeared in the Journal of Comparative Medicine and Veterinary Archives explaining the conditions, and its cause, of the disease which I saw fit to call "Millet-disease." At that time I did not deem it expedient to make reply to these critics, thinking that at a later date, when I had further experimented with millet, I could add something new on that subject.

The first article I wish to take up is that of G. J. Bowker, V.S., of Groton, N. Y., which will be found on page 9, No. 1, vol. xv., of the Journal. While this does not bear directly upon the subject under consideration, it is an indirect hint as to the cause of the disease. I quote the following: "Last summer I was in North Dakota, near the Red River Valley country, which is noted for its alkaline water. The water is so thoroughly impregnated with the salts of lime that in the summer the shirts of the men working in the sun, drinking large quantities of water and perspiring freely, will be all white from the sediment left after the sweat has been dried up."

What is known as alkaline water is alkaline because it contains sulphate of magnesia and sulphate of sodium, and not on account of the lime. The water of the Red River Valley contains but little lime, as will be seen by the following analysis:

| | Medora. | Fargo. | Jamestown. | Moorhead (artesian). | Kindred. | Devil's Lake. |
|---|---|---|---|---|---|---|
| | Parts per million. | Parts per million. | Parts per million. | Parts per million. | Parts per million. | Parts per million. |
| Total solids, | 1540 | 361 | 1300 | 637 | 1853 | 3476 |
| $SiO_2$ | Trace. | 5.71 | Trace. | Trace. | Trace. | Trace. |
| K.O | 2.07 | 1.97 | 2.85 | 7.03 | 12.03 | 7.68 |
| $Na_2O$ | 39.93 | 24 64 | 14.63 | 40.83 | 50.57 | 42.18 |
| Ca.O | Trace. | 21.53 | 27.25 | 10.68 | 1.73 | 0.80 |
| MgO | Trace. | Trace. | 7.18 | Trace. | 5.14 | Trace. |
| $FaO_3$ | 0 | 0 | Trace | 0 | Trace. | Trace. |
| $AlO_3$ | 0 | 0 | Trace. | 0 | Trace. | Trace. |
| $P_2O_5$ | 0.08 | 0 | Trace. | 0 | 0 | Trace. |
| $SO_3$ | 46.35 | 15.42 | 29.72 | 23.17 | 20.46 | 23.96 |

The most highly alkaline water of the above group is that from Medora, which shows 39.93 parts per million of soda. This is in the form of sulphate of soda or Glauber's salts. We know from personal experience that two glasses of that water are sufficient to act as a purgative.

Just think of the men sweating lime until their clothes are saturated, and enough has passed through that they become white on the outside! We think Dr. Bowker must have meant that as a joke, for if he ever studied physiology he would know better than to make an assertion of that kind, that the sweat-pores would give off such quantities of lime as that. By calculating from the above analysis he would see that a man would have to drink at least a barrel of water every twenty-four hours in order to get sufficient lime to saturate the outside of his clothes. The above analysis proves that his lime-theory is not well founded.

On page 41, vol. xv., is an article headed "Osteoporosis Not Millet-disease," by W. H. Mattson, V.M.D., in which Dr. Mattson attempts to argue the case without any knowledge whatever of its merits. I will take up his argument by beginning with the last proposition, in which he says: "I (Mattson) have fed millet to my horses for weeks together, and have known horses all around me to have been fed on it with no hay at all, and I have never yet seen any bad effects from it." Therefore, because Dr. Mattson and his neighbors have had no bad effects from millet, the millet produces no bad results. His argument falls flat. There are many people in the United States who have owned horses for years, who have never had a case of pneumonia, therefore horses do not have pneumonia, following out his line of argument. Dr. Mattson further says that my experiments seem to him a waste of time, as they show nothing whatever. "Things are not always what they seem." I am very sorry the doctor has no use for experiments, and that he can see no good come from them. I had hoped that every veterinarian would at least contribute some definite knowledge that usually can only be obtained by experimentation. He asked the question, "What has ventilation to do with the result of millet as food for horses?" It has much to do, if, as I maintain, its use produces the condition which we have outlined and have shown does exist in sections where that feed is used largely as coarse food. Thorough ventilation is a very important factor in the use of any food, and, when the tendency is for any food to act on the kidneys, ventilation should be of such a nature as to cause the freest action of the skin, and relieve those organs in that manner of as much work as possible.

The continued experiments here at the Station, from the feeding of millet, resulted in the total destruction of one animal and the laming of another for life, the animal now being entirely unfit for service of any kind. Post-mortem examinations have shown us that the synovial fluid in the joints, although there was no rupture in a single case, contained red blood-corpuscles in sufficient quantities to render it of a dark-blood color. An examination of the kidneys by Dr. Formad showed that chronic inflammation was present, and that other changes had taken place showing a tendency to fatty degeneration. These conditions we *know* were present after feeding millet for a period of about three months. We also know that the animal, as far as physical diagnosis was able to demonstrate, was entirely well at the time the experiment was begun. This animal, aged, was positively known not to have been sick for five years previously to using her for the experiment.

Before Dr. Mattson attempts to show us that this millet-disease is osteoporosis, we would like to have him publish a clear, clean-cut statement of what osteoporosis is, the pathological conditions that are present, and the cause of the same, as far as he has ascertained. If he does that, then we will be able to enter into a further discussion of the merits of this case.

T. D. HINEBAUCH.

FARGO, N. D.

---

New Zealand has prepared a bill for submission to the people looking to the exclusion of consumptives from their colony, from any country, under a very heavy penalty.

